# Case report: State-of-the-art risk-modifying treatment of sudden cardiac death in an asymptomatic patient with a mutation in the SCN5A gene and a review of the literature

**DOI:** 10.3389/fcvm.2023.1193878

**Published:** 2023-09-06

**Authors:** Petar Brlek, Eduard Stjepan Pavelić, Jana Mešić, Kristijan Vrdoljak, Andrea Skelin, Šime Manola, Nikola Pavlović, Jasmina Ćatić, Gordana Matijević, Josep Brugada, Dragan Primorac

**Affiliations:** ^1^ St. Catherine Specialty Hospital, Zagreb, Croatia; ^2^ School of Medicine, Josip Juraj Strossmayer University of Osijek, Osijek, Croatia; ^3^ Department for Cardiovascular Diseases, University Hospital Dubrava, Zagreb, Croatia; ^4^ Cardiovascular Institute, Hospital Clinic, University of Barcelona, Barcelona, Spain; ^5^ Faculty of Dental Medicine and Health, Josip Juraj Strossmayer University of Osijek, Osijek, Croatia; ^6^ Medical School, University of Rijeka, Rijeka, Croatia; ^7^ Medical School, University of Mostar, Mostar, Bosnia and Herzegovina; ^8^ Medical School, University of Split, Split, Croatia; ^9^ Department of Biochemistry & Molecular Biology, The Pennsylvania State University, State College, PA, United States; ^10^ The Henry C. Lee College of Criminal Justice and Forensic Sciences, University of New Haven, West Haven, CT, United States; ^11^ Medical School REGIOMED, Coburg, Germany; ^12^ National Forensic Sciences University, Gujarat, India

**Keywords:** Brugada syndrome, sudden cardiac death, SCN5A, implantable cardioverter-defibrillator, atrial fibrillation

## Abstract

Brugada syndrome is a rare hereditary disorder characterized by distinct ECG findings, complex genetics, and a high risk of sudden cardiac death. Recognition of the syndrome is crucial as it represents a paradigm of sudden death tragedy in individuals at the peak of their lives. Notably, Brugada syndrome accounts for more than 20% of sudden cardiac deaths in individuals with structurally normal hearts. Although this syndrome follows an autosomal dominant inheritance pattern, it is more prevalent and severe in males. Diagnosis is primarily based on the characteristic ECG pattern observed in the right precordial leads. Mutations in the SCN5A gene, resulting in loss of function, are the most common genetic cause. We presented a 36-year-old proband with a family history of sudden cardiac death. Although the patient was asymptomatic for Brugada syndrome, his father had experienced sudden death at the age of 36. The proband was admitted to St. Catherine's Specialty Hospital where blood was taken and subjected to next-generation sequencing (NGS) using a “Sudden cardiac death” panel. The analysis identified a pathogenic variant in the SCN5A gene [c.4222G > A(p.Gly1408Arg)], which is associated with autosomal dominant Brugada syndrome. Based on the positive genetic test result, the patient was referred for further examination. ECG with modified precordial lead positioning confirmed the presence of the Brugada phenotype, displaying the type-2 and type-1 ECG patterns. Therefore, we made the diagnosis and decided to implant an implantable cardioverter-defibrillator (ICD) based on the results of broad genetic NGS testing, diagnostic criteria (ECG), and considering the high burden of sudden cardiac death in the patient's family, as well as his concerns that limited his everyday activities. This case shows that genetics and personalized medicine hold immense potential in the primary prevention, diagnosis, and treatment of Brugada syndrome and sudden cardiac death.

## 1. Introduction

In an individual with no underlying condition that may appear fatal, an unexpected natural death from a cardiac cause (approximately within one hour after the onset) is defined as sudden cardiac death (1). This sudden onset accounts for up to 50% of cardiac deaths, and its occurrence decreases with age. Also, the etiology of sudden cardiac death changes with age. In pediatric and adolescent populations, the most common causes are coronary anomalies, hypertrophic cardiomyopathy, and myocarditis; while in adults, coronary atherosclerosis and acquired forms of cardiomyopathy are the most frequent findings at autopsies (2). Also, the cause for sudden cardiac death can be a primary electrical abnormality if the patient has no structural heart disease, such as Brugada syndrome (BrS) or long-QT syndrome (3).

BrS is a condition with an increased risk for abnormal heart activity to occur. It is an autosomal dominant inherited condition, and the gene that is most commonly affected is SCN5A. It encodes the cardiac sodium channel (4). The reported incidence of BrS varies between 5 and 30 per 10,000 people. Approximately 25% of those who screen positive for the disease also have a family member with the condition (5, 6). Patients who are symptomatic may experience episodes of syncope or even abnormal heart rhythms, including polymorphic ventricular tachycardia or ventricular fibrillation, which can lead to sudden cardiac death (7, 8). The episodes of fainting, despite a normal heart rhythm, are attributed to vasovagal syncope, which is characterized by a sudden decrease in blood pressure. Also, the symptoms and severity of BrS may vary even among family members with the same genetic variant (7). Electrocardiogram (ECG) may show a pseudo-right bundle branch block (RBBB) and typical “coved type” ST-segment elevation in the right precordial leads V1-V2. This elevation measures ≥2 mm (≥0.2 mV) and may occasionally be observed in the inferior leads as well (9).

Based on ECG patterns, three types of Brugada pattern have been classified. Type-1 Brugada pattern presents with a pathognomonic coved ST-segment elevation ≥ 2 mm followed by a negative T-wave, with little or no isoelectric separation. This characteristic presentation is observed in more than one right precordial lead, ranging from V1 to V3. On the other hand, Type-2 also consists an ST-segment elevation, but it is accompanied by a positive or biphasic T-wave, resulting in a saddle-back configuration. Type-3, in contrast to the previous two types, is characterized by a right precordial ST-segment elevation ≤ 1 mm. This elevation can be either in a coved-type or saddle-back morphology (10). However, only the type-1 pattern is deemed diagnostically significant (11).

The pathophysiology of the BrS has been associated with mutations in genes (23 genes to date) for sodium (Na+), calcium (Ca2+), or potassium (K+) ion channels in the cardiac cell membrane. They cause a decrease in inward Ca2 + or Na + currents or an increase in the outward K + currents. This results in a pro-arrhythmic outward shift in the balance of transmembrane currents during the early phase of the action potential (7). Out of these mutations, most of them occur in SCN5A (11–28%), SCN10A (16,7%), CACNA1C (6,6%), CACNB2b (4,8%), KCNJ8 (2%), CACNA2D1 (1,8%), and SCN1B (1,1%) (4). Regardless of all the above-mentioned symptoms and signs, most patients are asymptomatic. Although asymptomatic, these patients are still at risk of sudden cardiac death. Currently, no agreement has been found on the risk stratification approach for them (12). Increased risk of ventricular tachyarrhythmias (VT) may result in a fatal outcome in seemingly healthy individuals (9). Nevertheless, current genetic analysis can preventively diagnose BrS in asymptomatic patients and successfully treat them with innovative methods. ICD implantation remains the state-of-the-art treatment in high-risk patients (12).

This study aimed to present patient with a structurally normal heart whose genetic diagnostics led to preventive ICD implantation due to the Brugada syndrome family history.

## 2. Case description

A 36-year-old patient (proband) was admitted to St. Catherine's Specialty Hospital due to a repeated occurrence of sudden cardiac deaths in the family history. The patient has been regularly going to cardiologic check-ups and measurements (echocardiogram, heart stress test, 24-hour ECG monitoring). He has never been of symptoms that are characteristic of BrS. The patient underwent extensive clinical examination, including a cardiologist physical examination, ECG, ajmaline test (ECG following sodium channel blockage provocation), cardiac stress test, echocardiographic imaging, magnetic resonance imaging of the heart, and laboratory testing, including next-generation sequencing (NGS).

At 17 years of age, the patient reported fatigue after which he underwent ECG. The recording showed a right bundle branch block (RBBB) and left anterior hemiblock (LAHB). Since then, he has been going to regular cardiologic check-ups with a cardiac stress test (every year) which showed no signs of abnormal heart function. In September 2017, an ajmaline test was performed to reveal the ECG changes, and the patient tested negative for this sodium ion channel blockage provocation. 24-hour ECG imaging done in September 2021 showed a wide QRS complex typical for RBBB without other abnormalities. Following, in October 2021, due to a recent COVID-19 infection and palpitations, magnetic resonance imaging (MRI) of the heart was performed. Left ventricle diameter was at the upper normal limit (57 mm) and asynchronous contractions of the septum were found due to the RBBB. It showed no fibrosis. The patient underwent another ECG in November 2021, confirming RBBB and LAHB. Furthermore, a cardiac stress test was performed which was normal.

**Figure 1:**
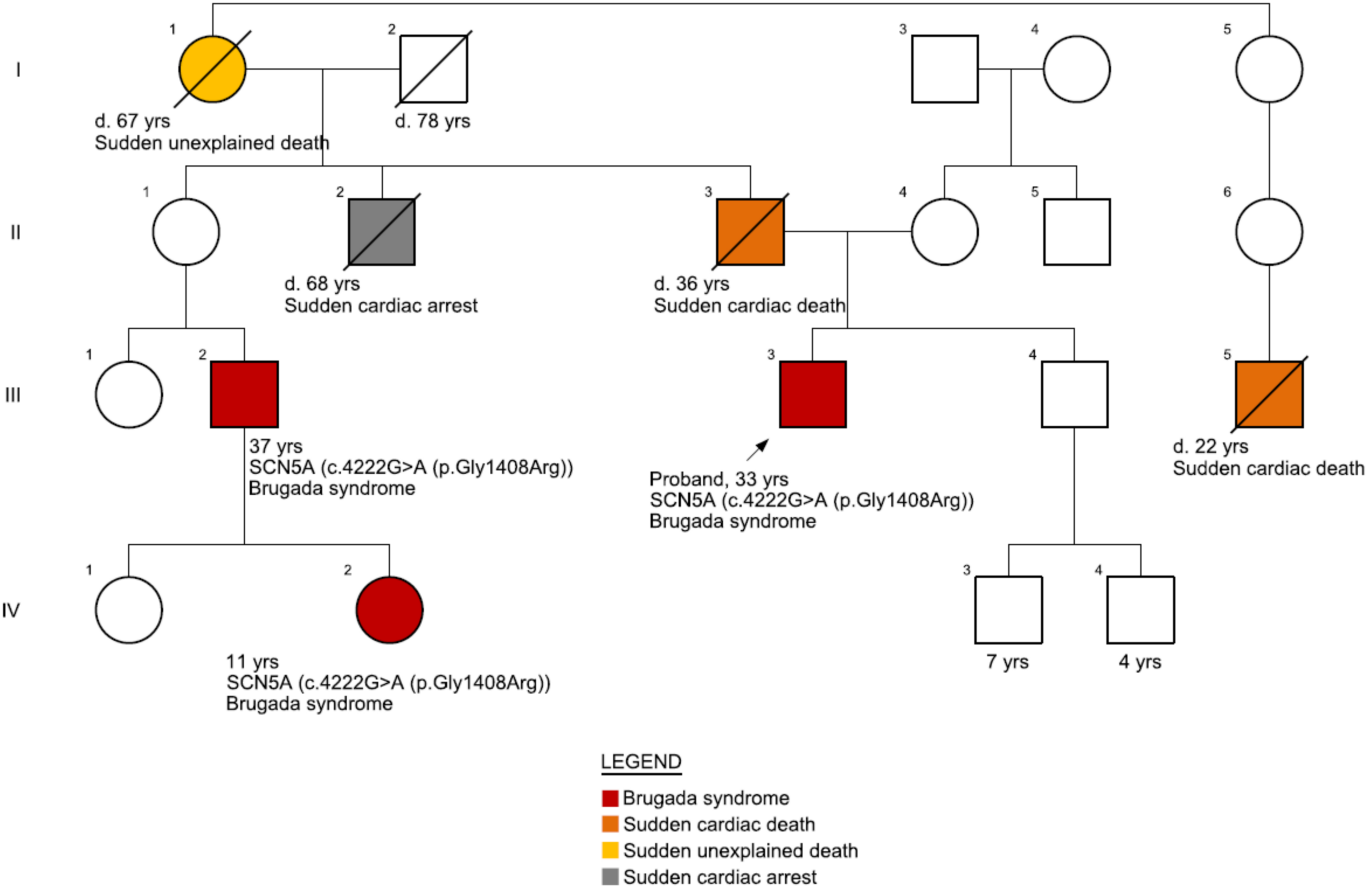
Family pedigree. The patient's father (II-3) suddenly passed away at 36 years of age with a suspected cardiac cause. His father's nephew (III-2) had suffered heart arrest at 37 years of age and was implanted with an implantable cardioverter-defibrillator (ICD) after he tested positive for BrS [pathogenic variant, SCN5A c.4222G > A (p.Gly1408Arg)]. The nephew's daughter (IV-2) also tested positive for BrS on genetic testing. Another close relative (III-5, grandson from his grandmother's sister) passed away at 22 years of age while playing basketball.

The patient's clinical presentation has been asymptomatic for the BrS condition. However, there is a positive BrS family history (Figure 1) as well as a repeated occurrence of sudden cardiac deaths. Family history revealed that the patient's father had suddenly passed away at the age of 36, with a suspected cardiac cause. Additionally, the patient's paternal grandmother had died at the age of 67, and the cause was unspecified. Furthermore, the patient's second cousin experienced cardiac arrest at the age of 38 and had an implanted cardioverter defibrillator (ICD) after testing positive for the pathogenic variant of the SCN5A gene, which is associated with Brugada syndrome. Moreover, the grandson of the patient's maternal grandmother's sister suddenly passed away at the age of 22 while playing basketball. The patient's uncle (father's brother) died at 68 years of age following hospitalization for two weeks after being diagnosed with tachycardia. The uncle had also suffered from several episodes of syncope during his lifetime. The patient's brother's ECG did not show any abnormalities, and echocardiogram control showed no pathology characteristic for BrS.

## 3. Timeline

Figure 2.

**Figure 2:**
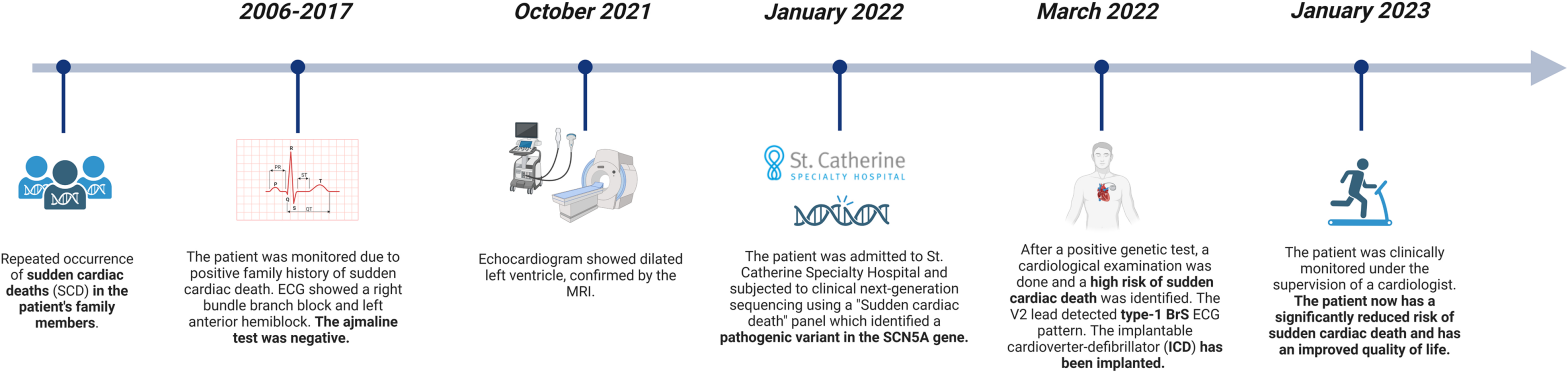
Timeline.

## 4. Diagnostic assessment

### 4.1. Genetic testing

Genomic DNA was isolated from the patient's blood sample and subjected to clinical next-generation sequencing using a “Sudden cardiac death” panel (13). The analysis included sequence analysis of 294 genes (Supplementary Table S1) which were sequenced to a minimum depth of ≥50× and an average of 350×. Sequence reads were aligned with the reference genome (GRCh37). Promoters and other non-coding regions were not included in this analysis. As described in our previous study, bioinformatics software Align-GVGD, Polymorphism Phenotyping v2 (PolyPhen-2), and Sorting Intolerant to Tolerant (SIFT) were used in order to determine the potential pathogenicity of the gene variants identified in proband's sample (14).

### 4.2. Genetic findings and implantation of cardioverter defibrillator

A comprehensive analysis using a multi-gene panel for “Sudden cardiac death” (as shown in Supplementary Table S1) detected a pathogenic variant in the SCN5A gene associated with a spectrum of autosomal dominant cardiac conditions including autosomal dominant Brugada syndrome (BrS), atrial fibrillation, dilated cardiomyopathy (DCM), and long QT syndrome (LQTS), type 3. The patient was heterozygous for the pathogenic variant of the SCN5A gene (c.4222G > A), which replaces glycine, which is neutral, non-polar and the smallest amino acid, with arginine, which is basic and polar, at codon 1,408 of the SCN5A protein (p.Gly1408Arg). The bioinformatics algorithms developed to evaluate the impact of missense changes on protein structure and function, including SIFT, PolyPhen-2, and Align-GVGD, unanimously indicated that this variant is highly likely to cause functional disruption. According to the American College of Medical Genetics and Genomics (ACMG) classification, the SCN5A c.4222G > A (p.Gly1408Arg) variant was classified as a pathogenic variant. Additionally, we identified one pathogenic (low penetrance) variant in the HFE gene associated with autosomal recessive hereditary hemochromatosis (HFE-HH) and 3 heterozygous variants of uncertain significance in genes ABCC9 [c.2224G > C (p.Glu742Gln)], COL12A1 [c.3188T > C (p.Ile1063Thr)], PLEC [c.2821-3C > A (Intronic)]. The patient is a carrier of autosomal recessive HFE-HH, which does impact reproductive risk (biological relatives have a chance of being at risk for autosomal recessive HFE-HH) but is insufficient to cause autosomal recessive HFE-HH in our patient.

**Figure 3:**
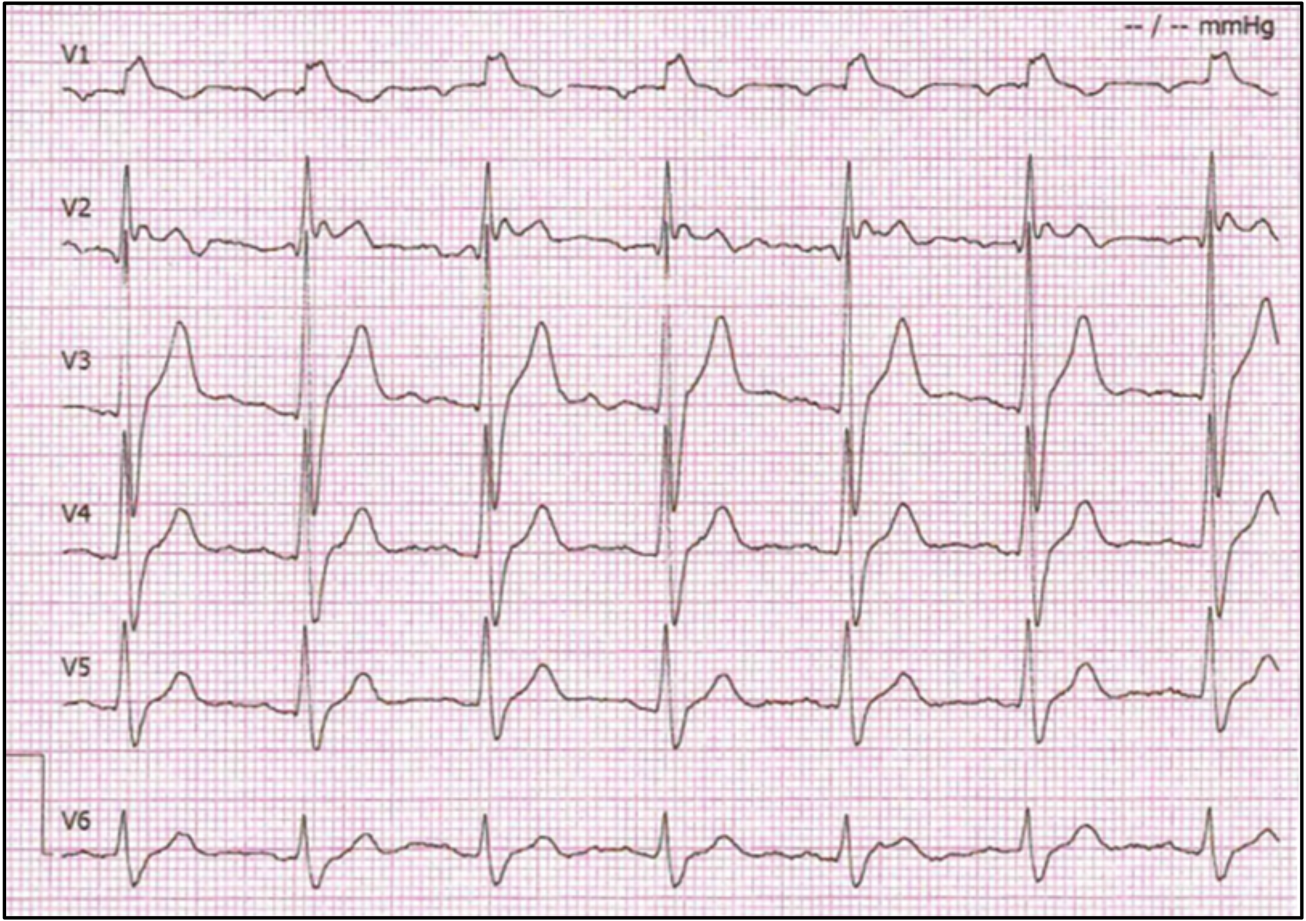
Proband's electrocardiogram. Precordial leads show right bundle branch block morphology with Brugada pattern ST segment elevation in V1 and V2. ECGs in this study were recorded at standard ECG paper speeds of 25 mm/s and 10 mm/mV.

Due to the positive result of the genetic test, the patient was referred for additional cardiology workup and treatment. The patient underwent ECG with modulated positioning of precordial leads to increase the sensitivity of the method for Brugada phenotype detection. The V2 lead detected a “saddle-back” ST-elevation that is characteristic of the type-2 BrS ECG pattern in majority of ECG strips, however one ECG showed typical type-1 BrS ECG pattern (Figure 3). Although the patient had no indication for an ICD implant according to current ESC Guidelines for prevention of sudden cardiac death since he has no history of ventricular arrhythmias, or arrhythmogenic syncope, due to the high burden of sudden cardiac death in his family as well as patient's concerns which limited him in everyday activities, the decision to implant an ICD was made. On March 22, 2022, ICD was implanted, which improved his quality of life.

## 5. Discussion

Brugada syndrome is a rare genetic condition characterized by ST-segment elevation in the right precordial electrocardiogram leads and a high risk of life-threatening ventricular arrhythmias. The prevalence of Brugada syndrome is only 5:10.000, but considering its deadly potential, recognizing the symptoms and signs of Brugada syndrome is crucial (15). Male gender is associated with a higher prevalence of BrS and ventricular arrhythmias at diagnosis and follow-up (12). A recent study shows that coding and non-coding genetic variants can modulate the transcriptional regulation of genes for voltage-gated sodium channels and impact conduction velocities and susceptibility to arrhythmias (16). The SCN5A gene loss-of-function mutations are the most common genotypes and have been found in Brugada syndrome Type 1, resulting in a weakened sodium current (INa) and are associated with dilation and impairment in contractile function of both ventricles (17). The gain-of-function mutations in the SCN5A gene result in an increased influx of sodium ions and leads to a condition called Long QT syndrome (LQTS) Type 3 (18). In addition, it's important to emphasize that SCN5A mutations can result in a spectrum of arrhythmogenic syndromes, often inherited as an autosomal dominant trait, characterized by incomplete penetrance, and a higher incidence in males. This complexity can lead to the presentation of overlap syndromes, potentially involving manifestations of sick sinus syndrome (SSS), progressive cardiac conduction disease (PCCD), Brugada syndrome (BrS), and type 3 long QT syndrome (LQTS) (19). Patients with LQTS type 3 exhibit a heightened susceptibility to experiencing arrhythmic events, particularly during physical exercise. Understanding the true risk of these adverse events in LQTS population may be extremely complex and potentially dependent on the affected gene, degree of QT prolongation, age, sex, and other yet unknown factors (20). This increased risk during exercise places them in a more precarious situation compared to Brugada syndrome patients who engage in similar physical activities (21). It leads us to question if Brugada syndrome is only a channelopathy or if it could be cardiomyopathy. Originally Brugada syndrome was considered a channelopathy without the presence of structural changes. However, recent evidence suggests that microanatomical changes in structures as proliferated collagen or fibrosis and even decreased gap junction expression, could lead to arrhythmic events. The recent study demonstrating the normalization of the ECG pattern after radiofrequency ablation of the RVOT epicardium suggests an important role of structural pathophysiology (12).

Approximately two-thirds of SCN5A pathogenic mutations are missense mutations, and the other one-third are non-missense mutations (22). More than 20 genes with an autosomal dominant inheritance of pathogenic variants have been discovered to this date. In 2007 the GPD1L gene loss-of-function mutation was discovered with a rare phenotype of Brugada syndrome Type 2 (23). Over the coming years, discoveries of the CACNA1C, CACNB2b, and SN1B gene mutations, causing Brugada syndrome Type 3, 4, and 5, have been published (24). Although more than 20 genes have been found, 80% of genotype-positive Brugada syndrome patients have a mutation in SCN5A (25). However, still 70–75% of patients with Brugada syndrome have an unknown genetic background (26).

Given that our patient did not exhibit any symptoms of Brugada syndrome in their clinical presentation, the confirmed pathogenic variant in the SCN5A gene held preventive significance. SCN5A gene encodes the pore-forming ion-conducting *α*-subunit of the cardiac sodium channel (Nav1.5) (27). The protein encoded by the SCN5A gene plays a crucial role in initiating and propagating action potentials, ultimately influencing cardiac excitability and the conduction of electrical currents. Mutations in this gene can lead to impaired function of SCN5A, which can occur through various mechanisms such as reduced expression levels of SCN5A in the sarcolemma, production of defective Nav1.5 proteins, or alterations in the gating properties of the channel resulting in decreased INa (delayed activation or earlier/faster inactivation) (28). The genetics underlying Brugada syndrome is complex, and it appears that the condition arises from the interplay of multiple genes. As a result of these complex interactions, the penetrance of BrS is known to be variable. This variability explains the phenomenon where certain individuals within a family who carry a specific mutation may exhibit signs of BrS, while others with the same mutation may not display any symptoms (29). However, the confirmed genetic diagnosis enhances medical experts to pay close attention to conditions that may occur with pathogenic variants even in asymptomatic individuals or some individuals with other cardiologic conditions such as first-degree heart block and isolated cardiac conduction disease (22, 30, 31). The patients should treat fever with antipyretic drugs early, avoid drugs that may cause ST elevation in right precordial leads and avoid use of cannabis, cocaine and alcohol. Since cardiac rhythm is influenced by the electrolyte concentrations, occasional assessment of electrolyte balance should be done—especially Ca, K and Na. Quinidine use may be indicated to prevent ventricular arrhythmias (32). Preventive measures are recommended to mitigate the risk of sudden death caused by severe abnormal heart rhythms, including polymorphic ventricular tachycardia and ventricular fibrillation (33). Lastly, there are guidelines for ICD implant in primary and secondary prevention of sudden cardiac death for patients with BrS. Currently, patients with aborted cardiac arrest and documented spontaneous VT have Class I indication for ICD implant (34). The ICD is a well-established and effective intervention for the prevention of sudden cardiac death, playing a vital role in managing high-risk cardiac conditions. However, selecting the most suitable ICD for an individual patient is a complex and intricate process, involving considerations such as the patient's specific condition, risk profile, and potential complications associated with various ICD types. A recent study involving 258 patients has revealed no significant differences in inappropriate ICD therapies, device-related complications, or infections between the population of drug-induced type-1 BrS patients with subcutaneous-ICD and those with transvenous-ICD. Furthermore, there's evidence suggesting that the use of subcutaneous-ICD may potentially reduce the risk of complications related to lead in patients with ICDs (35).

On the other hand, primary prevention indications are relatively “narrow” in patients with BrS and include a history of arrhythmic syncope (IIa) and patients with VF induced during an electrophysiologic study (IIb). Recent scientific findings offer compelling evidence that electrophysiologic study (EPS) serves as a valuable tool in risk stratification for patients with BrS. EPS facilitates the identification of individuals within the BrS population who might be suitable candidates for primary prevention of SCD through ICD implantation (36, 37). A retrospective analysis of a cohort of BrS patients, primarily conducted for preventive purposes, at five Italian medical centers has highlighted the significance of heterogeneity in the refractory period of the right ventricle as a critical factor in risk stratification for life-threatening arrhythmias, particularly within the BrS patient population (38). This observation underscores the potential to enhance the precision of prognostic assessments in asymptomatic patients with an SCN5A mutation.

Brugada syndrome has incomplete penetrance and variable gene expression, which complicates clinical diagnosis (39). The ECG is still the starting point in diagnosing Brugada syndrome, even though some patients are asymptomatic. For that reason, genetic diagnostics in asymptomatic people with a positive family history have a great impact on the identification of patients at risk. Other forms of inheritance patterns have been suggested, such as X-linked (KCNE5 gene) and autosomal recessive (TRPM4 gene) (40). Brugada syndrome was often considered a monogenic disease, but recent studies have shown its oligogenic and polygenic inheritance pattern with gene interactions stimulating or reducing phenotypic expression (3, 5, 17, 23). Therefore, the genetic basis of Brugada syndrome is heterogeneous, and no genotype can currently be determined in most patients. However, recent studies suggest that mutations of the SCNA5 gene that affect pore region are associated with severe phenotype and greater risk of adverse cardiac events in asymptomatic as well as in symptomatic patients (12). Next-generation sequencing (NGS) enables the detection of pathogenic mutations in the genes responsible for all forms of Brugada syndrome. In our NGS panel, it is possible to determine gene variants in 294 genes associated with sudden cardiac death, and such multi-gene testing is the only way to detect individuals at risk for sudden cardiac death in asymptomatic patients.

Nevertheless, the future of genetics and personalized medicine is bright, and it could noticeably increase the significance of genetic testing, especially in terms of primary prevention, diagnosis, and treatment of patients with sudden cardiac death.

## 6. Patient perspective

“After the implantation of an implantable cardioverter defibrillator, I continued to play football and basketball and run and train regularly. The information regarding my positive test for a pathogenic variant of the SCN5A gene is critical for my future and family planning, as I am now aware of the potential risks. I was burdened and afraid because of the sudden cardiac deaths in my family and I feel safer with implantable ICD now. Finally, I can lead a normal life”.

## Data Availability

The original contributions presented in the study are included in the article/**Supplementary Material**, further inquiries can be directed to the corresponding author.
