# Supplementary material for: Case report: State-of-the-art risk-modifying treatment of sudden cardiac death in an asymptomatic patient with a mutation in the SCN5A gene and a review of the literature
Source: Front Cardiovasc Med. 2023 Sep 6;10:1193878. doi: 10.3389/fcvm.2023.1193878 (PMC10512029; doi:10.3389/fcvm.2023.1193878)
Supplement: Supplementary file 1 [file Table1.docx]

Supplementary Material

# Supplementary Table

**Table 1.** A complete list of genes analyzed, including the relevant gene transcripts.

| GENE | TRANSCRIPT | GENE | TRANSCRIPT | GENE | TRANSCRIPT | GENE | TRANSCRIPT |
| --- | --- | --- | --- | --- | --- | --- | --- |
| A2Ml1 | NM_144670.4 | B4GAT1 | NM_006876.2 | CHD7 | NM_017780.3 | DEPDC5 | NM_001242896.1 |
| ABCC9 | NM_005691.3 | BAG3 | NM_004281.3 | CHKB | NM_005198.4 | DES | NM_001927.3 |
| ABCG5 | NM_022436.2 | BCOR | NM_017745.5 | CHRM2 | NM_000739.2 | DMD | NM_004006.2 |
| ABCG8 | NM_022437.2 | BIN1 | NM_139343.2 | CHST14 | NM_130468.3 | DNAJC19 | NM_145261.3 |
| ACADVL | NM_000018.3 | BMPR1B | NM_001203.2 | CNTN1 | NM_001843.3 | DNM2 | NM_001005360.2 |
| ACTA1 | NM_001100.3 | BMPR2 | NM_001204.6 | COL12A1 | NM_004370.5 | DOLK | NM_014908.3 |
| ACTA2 | NM_001613.2 | BRAF | NM_004333.4 | COL1A1 | NM_000088.3 | DPM1 | NM_003859.1 |
| ACTC1 | NM_005159.4 | CACNA1C | NM_000719.6; NM_001129840.1 | COL1A2 | NM_000089.3 | DPM2 | NM_003863.3 |
| ACTN2 | NM_001103.3 | CACNA2D1 | NM_000722.3 | COL3A1 | NM_000090.3 | DPM3 | NM_153741.1 |
| ACVR2B | NM_001106.3 | CACNB2 | NM_201590.2 | COL5A1 | NM_000093.4 | DSC2 | NM_024422.4 |
| ACVRL1 | NM_000020.2 | CALM1 | NM_006888.4 | COL5A2 | NM_000393.3 | DSG2 | NM_001943.3 |
| ADAMTS2 | NM_014244.4 | CALM2 | NM_001743.4 | COL6A1 | NM_001848.2 | DSP | NM_004415.2 |
| AGL | NM_000642.2 | CALM3 | NM_005184.2 | COL6A2 | NM_001849.3 | DTNA | NM_001390.4 |
| AKAP9 | NM_005751.4 | CALR3 | NM_145046.4 | COL6A3 | NM_004369.3 | DYSF | NM_003494.3 |
| ALMS1 | NM_015120.4 | CAPN3* | NM_000070.2 | CPT2 | NM_000098.2 | EFEMP2 | NM_016938.4 |
| ANK2 | NM_001148.4 | CASQ2 | NM_001232.3 | CRELD1 | NM_001031717.3 | ELAC2 | NM_018127.6 |
| ANKRD1* | NM_014391.2 | CAV1 | NM_001753.4 | CRTAP | NM_006371.4 | ELN | NM_001278939.1 |
| ANO5 | NM_213599.2 | CAV3 | NM_033337.2 | CRYAB | NM_001885.2 | EMD | NM_000117.2 |
| APOB | NM_000384.2 | CBL | NM_005188.3 | CSRP3 | NM_003476.4 | ENG* | NM_000118.3 |
| ATP2A1 | NM_173201.3 | CBS | NM_000071.2 | CTF1* | NM_001330.3 | EYA4 | NM_004100.4 |
| ATP7A | NM_000052.6 | CCDC78 | NM_001031737.2 | CTNNA3 | NM_013266.3 | FBN1 | NM_000138.4 |
| B3GALNT2 | NM_152490.4 | CFL2 | NM_021914.7 | DAG1 | NM_004393.5 | FBN2 | NM_001999.3 |
| FHL1 | NM_001449.4 | ITGA7 | NM_002206.2 | LIMS2 | NM_001136037.2 | NKX2-5 | NM_004387.3 |
| FHL2 | NM_201555.1 | JAG1 | NM_000214.2 | LMNA | NM_170707.3 | NKX2-6 | NM_001136271.2 |
| FKBP14 | NM_017946.3 | JPH2 | NM_020433.4 | LMOD3 | NM_198271.4 | NODAL | NM_018055.4 |
| FKRP | NM_024301.4 | JUP | NM_002230.2 | LRRC10 | NM_201550.3 | NOTCH1 | NM_017617.3 |
| FKTN | NM_001079802.1 | KBTBD13 | NM_001101362.2 | MAP2K1 | NM_002755.3 | NPPA | NM_006172.3 |
| FLNA | NM_001456.3 | KCNA1 | NM_000217.2 | MAP2K2 | NM_030662.3 | NR2F2 | NM_021005.3 |
| FLNC* | NM_001458.4 | KCNA5 | NM_002234.3 | MAT2A | NM_005911.5 | NRAS | NM_002524.4 |
| FOXE3 | NM_012186.2 | KCND3 | NM_004980.4 | MATR3 | NM_199189.2 | NSD1 | NM_022455.4 |
| FOXH1 | NM_003923.2 | KCNE1 | NM_000219.5 | MED12 | NM_005120.2 | P3H1 | NM_022356.3 |
| GAA | NM_000152.3 | KCNE2 | NM_172201.1 | MED13L | NM_015335.4 | PCDH19 | NM_001184880.1 |
| GATA4 | NM_002052.3 | KCNE3 | NM_005472.4 | MEGF10 | NM_032446.2 | PCSK9 | NM_174936.3 |
| GATA6 | NM_005257.5 | KCNE5 | NM_012282.2 | MEIS2 | NM_170674.4 | PDLIM3 | NM_014476.5 |
| GATAD1 | NM_021167.4 | KCNH2 | NM_000238.3 | MTM1 | NM_000252.2 | PKP2 | NM_004572.3 |
| GDF1 | NM_001492.5 | KCNJ2 | NM_000891.2 | MTO1 | NM_012123.3 | PLEC | NM_000445.4; NM_201378.3 |
| GDF2 | NM_016204.2 | KCNJ5 | NM_000890.3 | MYBPC3 | NM_000256.3 | PLEKHM2 | NM_015164.2 |
| GJA1 | NM_000165.4 | KCNJ8 | NM_004982.3 | MYF6 | NM_002469.2 | PLN | NM_002667.3 |
| GJA5 | NM_005266.6 | KCNK3 | NM_002246.2 | MYH11 | NM_001040113.1 | PLOD1 | NM_000302.3 |
| GLA | NM_000169.2 | KCNQ1 | NM_000218.2 | MYH6 | NM_002471.3 | PNPLA2 | NM_020376.3 |
| GMPPB | NM_021971.2 | KCNQ2 | NM_172107.2 | MYH7 | NM_000257.3 | POMGNT1 | NM_017739.3 |
| GNE | NM_001128227.2 | KCNQ3 | NM_004519.3 | MYL2 | NM_000432.3 | POMGNT2 | NM_032806.5 |
| GPC3* | NM_004484.3 | KCNT1 | NM_020822.2 | MYL3 | NM_000258.2 | POMK | NM_032237.4 |
| GPD1L | NM_015141.3 | KLHL40 | NM_152393.3 | MYL4 | NM_001002841.1 | POMT1 | NM_007171.3 |
| GYS1 | NM_002103.4 | KLHL41 | NM_006063.2 | MYLK | NM_053025.3 | POMT2 | NM_013382.5 |
| HAMP | NM_021175.2 | KRAS | NM_004985.4 | MYLK2 | NM_033118.3 | PRDM16* | NM_022114.3 |
| HAND1 | NM_004821.2 | LAMA2 | NM_000426.3 | MYOM1 | NM_003803.3 | PRKAG2 | NM_016203.3 |
| HCN4 | NM_005477.2 | LAMA4 | NM_002290.4 | MYOT | NM_006790.2 | PRKG1 | NM_006258.3 |
| HFE | NM_000410.3 | LAMP2 | NM_002294.2 | MYOZ2 | NM_016599.4 | PRRT2 | NM_145239.2 |
| HJV | NM_213653.3 | LARGE1 | NM_004737.4 | MYPN | NM_032578.3 | PTPN11 | NM_002834.3 |
| HNRNPDL | NM_031372.3 | LDB3 | NM_001080116.1; NM_001171610.1; NM_007078.3 | NEB* | NM_001271208.1 | RAF1 | NM_002880.3 |
| HRAS | NM_005343.2 | LDLR | NM_000527.4 | NEBL | NM_006393.2 | RANGRF | NM_016492.4 |
| ILK | NM_004517.3 | LDLRAP1 | NM_015627.2 | NEXN | NM_144573.3 | RASA1 | NM_002890.2 |
| ISPD | NM_001101426.3 | LEFTY2 | NM_003240.3 | NF1* | NM_000267.3 | RBM20 | NM_001134363.2 |
| RIT1 | NM_006912.5 | SGCG | NM_000231.2 | STIM1 | NM_003156.3 | TNNT1 | NM_003283.5 |
| RRAS | NM_006270.4 | SHOC2 | NM_007373.3 | SUN1 | NM_001130965.2 | TNNT2 | NM_001001430.2 |
| RXYLT1 | NM_014254.2 | SKI | NM_003036.3 | SUN2 | NM_015374.2 | TNPO3 | NM_012470.3 |
| RYR1 | NM_000540.2 | SLC22A5 | NM_003060.3 | SYNE1 | NM_033071.3 | TOR1AIP1 | NM_001267578.1 |
| RYR2 | NM_001035.2 | SLC2A1 | NM_006516.2 | SYNE2 | NM_182914.2 | TPM1 | NM_001018005.1 |
| SCN10A | NM_006514.3 | SLC2A10 | NM_030777.3 | TBX1 | NM_080647.1 | TPM2 | NM_003289.3 |
| SCN1A | NM_001165963.1 | SLC39A13 | NM_152264.4 | TBX5 | NM_000192.3 | TPM3* | NM_152263.3 |
| SCN1B | NM_199037.3; NM_001037.4 | SLC40A1 | NM_014585.5 | TCAP | NM_003673.3 | TRAPPC11 | NM_021942.5 |
| SCN2B | NM_004588.4 | SLMAP | NM_007159.2 | TFR2 | NM_003227.3 | TRDN | NM_006073.3 |
| SCN3B | NM_018400.3 | SMAD3 | NM_005902.3 | TGFB2 | NM_003238.3 | TRIM32 | NM_012210.3 |
| SCN4B | NM_174934.3 | SMAD4 | NM_005359.5 | TGFB3 | NM_003239.3 | TRPM4 | NM_017636.3 |
| SCN5A | NM_198056.2 | SMAD6 | NM_005585.4 | TGFBR1 | NM_004612.2 | TTN* | NM_001267550.2 |
| SCN8A | NM_014191.3; NM_001330260.1 | SMAD9 | NM_001127217.2 | TGFBR2 | NM_003242.5 | TTR | NM_000371.3 |
| SCN9A | NM_002977.3 | SNTA1 | NM_003098.2 | TIA1 | NM_022173.2 | TXNRD2 | NM_006440.4 |
| SDHA* | NM_004168.3 | SOS1 | NM_005633.3 | TMEM43 | NM_024334.2 | VCL | NM_014000.2 |
| SELENON | NM_020451.2 | SOS2 | NM_006939.2 | TMEM70 | NM_017866.5 | VCP | NM_007126.3 |
| SEMA3E | NM_012431.2 | SPRED1 | NM_152594.2 | TMPO | NM_003276.2 | ZFPM2 | NM_012082.3 |
| SGCA | NM_000023.2 | SQSTM1 | NM_003900.4 | TNNC1 | NM_003280.2 | ZIC3 | NM_003413.3 |
| SGCB | NM_000232.4 | SMAD4 | NM_005359.5 | TGFB3 | NM_003239.3 |  |  |
| SGCD | NM_000337.5 | STAC3 | NM_145064.2 | TNNI3 | NM_000363.4 |  |  |
